# Real-time PCR assay in differentiating *Entamoeba histolytica*, *Entamoeba dispar*, and *Entamoeba moshkovskii* infections in Orang Asli settlements in Malaysia

**DOI:** 10.1186/1756-3305-6-250

**Published:** 2013-08-28

**Authors:** Yee Ling Lau, Claudia Anthony, Siti Aminah Fakhrurrazi, Jamaiah Ibrahim, Init Ithoi, Rohela Mahmud

**Affiliations:** 1 Tropical Infectious Disease Research and Education Center (TIDREC), Department of Parasitology, Faculty of Medicine, University Malaya, 50603 Kuala Lumpur, Malaysia; 2 Department of Parasitology, Faculty of Medicine, University Malaya, 50603 Kuala Lumpur, Malaysia

**Keywords:** Amebiasis, Orang Asli settlements, Nested PCR, Real-time PCR, Microscopy

## Abstract

**Background:**

Amebiasis caused by *Entamoeba histolytica* is the third leading cause of death worldwide. This pathogenic amoeba is morphologically indistinguishable from *E. dispar* and *E. moshkovskii*, the non-pathogenic species. Polymerase chain reaction is the current method of choice approved by World Health Organization. Real-time PCR is another attractive molecular method for diagnosis of infectious diseases as post-PCR analyses are eliminated and turnaround times are shorter. The present work aimed to compare the results of *Entamoeba* species identification using the real-time assay against the established nested PCR method.

**Methods:**

In this study, a total of 334 human faecal samples were collected from different Orang Asli settlements. Faecal samples were processed by direct wet smear and formalin ethyl acetate concentration methods followed by iodine staining and was microscopically examined for *Entamoeba* species and other intestinal parasites. Microscopically positive samples were then subject to nested PCR and real-time PCR.

**Results:**

The overall prevalence of *Entamoeba* infection was 19.5% (65/334). SK Posh Piah recorded highest *Entamoeba* prevalence (63.3%) while Kampung Kemensah had the lowest prevalence (3.7%) of *Entamoeba*. Microscopically positive samples were then tested by real-time PCR and nested PCR for the presence of *Entamoeba histolytica*, *Entamoeba dispar*, and *Entamoeba moshkovskii* infection. Real-time PCR showed higher *Entamoeba* detection (86.2%) compared to nested PCR (80%), although the McNemar test value showed no significant difference between the two methods (p = 0.221).

**Conclusions:**

This study is the first in Malaysia to report the use of real-time PCR in identifying and differentiating the three *Entamoeba* infections. It is also proven to be more effective compared to the conventional nested PCR molecular method.

## Background

Amebiasis is the third leading cause of death worldwide. It is responsible for up to 100,000 deaths annually [1]. There are many species in the genus *Entamoeba*, of which, *Entamoeba histolytica*, *Entamoeba dispar*, *Entamoeba moshkovskii*, *Entamoeba coli*, *Entamoeba polecki*, and *Entamoeba hartmanni* are found in the intestinal lumen of humans [2]. Both *E. dispar* and *E. histolytica* are able to colonize humans but only *E. histolytica* is able to bring about invasive disease [3]. *E*. *moshkovskii* is primarily considered to be a free-living ubiquitous amoeba found in anoxic sediments [4]. The recently discovered *E. bangladeshi*, although distinct was clearly grouped with the clade of *Entamoeba* infecting humans, including *E. histolytica*. However, *E. bangladeshi* was found to be more distantly related than *E. dispar* but closer than *E*. *moshkovskii,* to *E. histolytica*[5].

*Entamoeba* infections are traditionally diagnosed via microscopic examination of stool samples, fresh or fixed. The pathogenic amoeba, *E. histolytica* is indistinguishable in its cyst and trophozite stages from *E. dispar* and *E. moshkovskii*, the non-pathogenic species [6]. It has also been shown that the sensitivity and specificity of microscopy is less optimal in differentiating the various species of *Entamoeba*[7]. Given the discrepancies of microscopy, various approaches have been implemented. The epidemiology of *Entamoeba* can be further studied by culturing trophozoites and determining isoenzyme patterns by gel electrophoresis [8]. These techniques, however, are costly, time-consuming, laborious and not practical for regular diagnosis [9]. An enzyme immunoassay kit (TechLab II antigen test) has been specifically designed for the detection of *E. histolytica*. However, this kit is marketed for examination of stool samples only and it has also been noted that fixed stools samples are not suitable for enzyme-linked immunosorbent assay (ELISA) [10,11]. Due to the dire need to study the epidemiology of *Entamoeba*, the polymerase chain reaction (PCR) is now the method of choice and this technique has been approved by the World Health Organization (WHO) [12]. In a study conducted by Stark *et al*. [13], it was demonstrated that PCR has improved sensitivity and specificity over ELISA-based kits. PCR also has the ability of specifically targeting and detecting *E. histolytica*, *E. dispar*, and *E. moshkovskii* infections [13,14].

Real-time PCR is still its infancy but it is a very attractive methodology for laboratory diagnosis of infectious diseases because of a lack of requirement for post-PCR analysis, resulting in shorter turnaround times and minimizing the risk of amplicon contamination [14,15]. This reflects obvious advantages in diagnostics, as amplicon contamination has been identified to be the most frequent cause of false-positive results in PCR amplification [16]. Aside from that, real-time PCR is a quantitative method and enables the determination of the number of parasites in various samples [17].

In Malaysia, water-borne and food diseases which are closely linked with personal hygiene and sanitation practices as well as environmental factors still pose as prominent health problems in rural settlements in Malaysia, particularly among Orang Asli communities; Malaysian aborigines, which make up 0.6% of the total population [18]. A recent study carried out in rural areas of Malaysia showed that 10.2% of the participants were infected with *Entamoeba*[19]. Other local studies pertaining to aboriginal groups reported prevalence rates ranging from 9.4% to 18.5% but these rates were solely based on microscopic examination which is unable to differentiate between *E. histolytica*, *E. dispar*, and *E. moshkovskii* infections [20]. In order to avoid unnecessary treatment of individuals infected with other species of *Entamoeba*, it is of utmost importance to specifically diagnose these species rather than provide treatment based on microscopic examination of feces [21].

In this study, real-time PCR was used to differentiate *E. histolytica*, *E. dispar*, and *E. moshkovskii* infections from several villages in Malaysia. To the best of our knowledge, this is the first study in Malaysia whereby real-time PCR assay was used to discriminate between the three species. This study was also aimed at comparing results of *Entamoeba* species identification using the real-time assay against the established nested PCR method.

## Methods

### Study area

Faecal specimens were collected from 7 different Orang Asli settlements in Pulau Carey (Kampung Bumbun), Hulu Yam (Kampung Gurney), Hulu Langat (Kampung Pangsun and Kampung Kemensah), Bentong (Kampung Sungai Minyak) and Kuala Kangsar (SK Pos Piah and Kampung Teras Pos Piah) during the period of May 2010 until October 2011. A total of 334 faecal specimens from volunteers aged ≤ 1 year to ≥18 years old were used in this study.

### Consent, sample processing and microscopic examination

The protocol of this study was approved by the University of Malaya Medical Ethics Committee (Ethics reference number, 914.4). Prior to sample collection, participants were briefed in the Malay language by the investigator about the study. Oral consent was obtained from the participants of the study. After consent was given, pre-labelled plastic containers for stool collection were given out to the participants.

Containers containing fresh faecal samples were collected and stored at ambient temperature. Upon arrival at the laboratory, samples were preserved in 5% potassium dichromate to avoid fungal growth and for preservation of the cysts and oocysts of the protozoa. Samples were stored at 4°C until further analysis. A small amount of faecal material (sediment of the formalin ethyl acetate concentration technique) [22] was mixed with one drop of iodine on a clean and dry microscope slide. Then, the mixture was covered with a cover slip and was observed under low (10×) and high (40×) magnification for the presence of *Entamoeba* species and other intestinal parasites. Cysts in stool samples were determined based on their shape, size and the number of nuclei observed.

### DNA extraction

Faecal specimens (0.25 g) were used for DNA extraction. DNA was extracted using the Mo Bio Power Soil DNA Isolation Kit (Mo Bio Laboratories California, USA) according to the manufacturer’s instructions. The DNA was eluted in 30 μl C6 solution (10 mM Tris). Extracted DNA was stored at −20°C until further use.

### Nested PCR assay

This assay was based on the amplification of the small-subunit rRNA gene of *E. histolytica*, *E. dispar* and *E. moshkovskii*. The primary PCR for the detection of *Entamoeba* genus used forward primer, E-1 (5’-TAA GAT GCA GAG CGA AA-3’) and reverse primer, E-2 (5’-GTA CAA AGG GCA GGG ACG TA-3’). The PCR was performed in a 25 μl reaction containing 2.5 μl of 10× PCR buffer, 2 μl of 1.25 mM dNTPs, 1.5 μl of 25 mM MgCl_2_, 0.5 μl of 10 pmole of each primer, 0.25 μl of 2.5U of Taq polymerase and 2.5 μl of DNA template. Nuclease free water was added to a final volume of 25 μl. The reaction was carried out with an initial denaturing step at 96°C for 2 minutes, followed by 30 cycles of 92°C for 1 minute (denaturation), 56°C for 1 minute (annealing), 72°C (extension) for 90 seconds and a final extension for 7 minutes at 72°C.

Subsequently, the primary PCR products were put through a 2nd round of PCR for *Entamoeba* species-specific characterization. Amplification was carried out using the following primer sets: EH-1 (5’-AAG CAT TGT TTC TAG ATC TGA G-3’) and EH-2 (5’- AAG AGG TCT AAC CGA AAT TAG-3’) to detect *E. histolytica* (439 bp); ED-1 (5’- TCT AAT TTC GAT TAG AAC TCT-3’) and ED-2 (5’-TCC CTA CCT ATT AGA CAT AGC-3’) to characterize *E. dispar* (174 bp); Mos-1 (5’-GAA ACC AAG AGT TTC ACA AC-3’) and Mos-2 (5’-CAA TAT AAG GCT TGG ATG AT-3’) to identify *E. moshkovskii* (553 bp). The secondary amplification used the same concentration of reagents as the primary reaction except that 2.5 μl of the primary PCR product was used as template instead of genomic DNA. The cycling conditions for the secondary amplification were the same as the primary reaction except the annealing temperature which was lowered to 48°C [6]. The specificity of the nested PCR assay was tested against DNA extracted from faecal samples of other protozoans, namely *Escherichia coli*, *Blastocystis hominis*, *Giardia intestinalis*, and *Cryptosporidium* sp. A total of 25 parasite-free faecal samples were also used to test the specificity of the nested PCR assay. All the control DNA samples were subjected to the same amplification protocol.

The PCR products were analyzed on a 2% agarose gel, stained with SYBR Safe DNA and the average band densities of the PCR products were measured using Quantity One software (Bio-Rad).

### Real-time PCR assay

Real-time PCR for 18S rRNA of *E. histolytica*, *E. dispar* and *E. moshkovskii* was performed using the CFX96™ Real-Time System (Bio-RAD). The reverse primer, Ehd-88R (5’-GCGGACGGCTCATTATAACA-3’) and Taqman probes for *E. histolytica* (histolytica-96 T; FAM-5’ UCAUUGAAUGAAUUGGCCAUUU 3’-BHQ1) and *E. dispar* (dispar-96 T; HEX-5’ UUACUUACAUAAAUUGGCCACUUUG 3’-BHQ1) used were previously described by Qvarnstrom and co-workers (2005) [10]. The forward primer (EM-RT-F2; 5’-GTCCTCGATACTACCAAC-3’) and probe (Em-TR; TxRd-5’ CCGTGAAGAGAGTGGCCGAC 3’-BHQ2) of *E. moshkovskii* were designed manually. The forward and reverse primers are conserved in the three *Entamoeba* species in SSU rRNA sequences. Three probes, namely histolytica-96 T, dispar-96 T and Em-TR were specifically designed to bind internally to the amplification primers to identify and differentiate *E. histolytica*, *E. dispar*, and *E. moshkovskii* from other organisms.

Each species was differentiated by different coloured filters in real-time PCR. *E. moshkovskii* was identified and differentiated from *E. histolytica and E. dispar* by Em-TR probe binding, which can be seen in channel 3. *E. histolytica* and *E. dispar* are detected by labelled probe histolytica-96 T and dispar-96 T and this was monitored in channels 1 and 2 respectively. All primers, probes, and reaction conditions were optimized according to a standard protocol described for the CFX96™ Real-Time System (Bio-RAD).

Amplification reactions were performed in a total volume of 20 μl with 10 μl of Bio-Rad SsoFast™ Probe Supermix (contains dNTPs, Sso7d fusion polymerase, MgCl_2_ and stabilizers), 10 μM EM-RT-F2 and Ehd-88R primers, 10 μM of histolytica-96 T, dispar-96 T and Em-TR probes, 5.4 μl of distilled H_2_O and 2.0 μl of DNA sample.

The amplification program was carried out according to the following; initial denaturation step at 95°C for 2 minutes, followed by 40 cycles of denaturation for 15 seconds at 95°C and annealing/extension at 60°C for 30 seconds. Fluorescence was measured at the end of each annealing/extension step. Amplification results were analyzed using CFX Manager™ software, version 2.1 for CFX96™. A sample was considered positive if the signal cycle threshold (*C*_*T*_) value exceeded a present threshold.

The minimum number of parasites detectable (detection limit) by real-time PCR assay was determined by 2-fold serial dilutions performed on *Entamoeba* sp. positive samples. The parasite concentrations were based on the amount of cysts counted under microscopy in samples ranging from 10 cysts to 0.6 cysts.

The specificity of the real-time PCR assay was tested against DNA extracted from faecal samples, namely *Escherichia coli*, *Blastocystis hominis*, *Giardia intestinalis*, and *Cryptosporidium* sp. A total of 25 parasite-free faecal samples were also used to test the specificity of the real-time PCR assay. All the control DNA samples were subjected to the same amplification protocol. Cross-reaction or cross-amplification between the three *Entamoeba* species was tested with each species specific *Entamoeba* primers and probes.

### Statistical analysis

Results from the comparison of the conventional nested PCR against real-time PCR are shown in a 2 × 2 table (Table 1). Agreement of results between the two methods was assessed using the Cohen’s kappa test [23] for concordance and McNemar’s test for discordance.

**Table 1:** Comparison of real-time PCR and nested PCR assays

**Nested PCR result**	**RT-PCR result**		
	**Positive**	**Negative**	**Total**
Positive	51	1	52
Negative	5	37	42
Total	56	38	94

Total microscopy positive samples tested: 65, Cohen’s kappa = 0.88; McNemar *P* = 0.221.

## Results

### Prevalence of *Entamoeba* infection by microscopy

A total of 334 faecal samples were collected from the Orang Asli settlements and screened via microscopy. From this total, 65 (19.5%) samples were microscopically positive for *Entamoeba* cysts, either singly or in combination with other intestinal parasites. SK Posh Piah had highest *Entamoeba* prevalence (63.3%) followed by Kampung Bumbun (28%), Kampung Teras Pos Piah (19.1%), Kampung Pangsun (18.2%), Kampung Gurney (10.4%), Kampung Sungai Minyak (6.9%) and Kampung Kemensah (3.7%) with the lowest prevalence of *Entamoeba* (Table 2).

**Table 2:** **Prevalence of *Entamoeba *infection based on microscopy, nested PCR, and real-time PCR assays**

**Location**	**No.**	**Microscopy**		**Nested PCR**		**Real-time PCR**	
	**Examined**	**n**	**%**	**n**	**%**	**n**	**%**
Kampung Gurney	48	5	10.4	4	80	5	100
Kampung Bumbun	50	14	28	12	85.7	14	100
Kampung Pangsun	55	10	18.2	10	100	10	100
Kampung Sungai Minyak	29	2	6.9	2	100	2	100
Kampung Kemensah	54	2	3.7	2	100	1	50
SK Pos Piah	30	19	63.3	14	73.7	16	84.2
Kampung Teras Pos Piah	68	13	19.1	8	61.5	8	61.5
Total	334	65	19.5	52	80	56	86.2

### Nested PCR and real-time PCR

Of the 65 microscopically positive samples, 52 (80%) samples were successfully amplified by nested PCR and *Entamoeba* species characterized based on its amplicon size. Of these 52 samples, 34 (65.4%) were found to be *E. histolytica*, 7 (13.5%) were identified as *E. dispar* and none were identified as *E. moshkovskii*. There were 11 (21.2%) samples which had a mixed infection of *E. histolytica* and *E. dispar* (Table 3).

**Table 3:** **Prevalence of *E. histolytica*, *E. dispar*, and *E. moshkovskii *infections based on PCR in microscopically positive faecal samples according to locations**

**Location**	**Nested PCR**	***E. histolytica***		***E. dispar***		***E. moshkovskii***		***E. histolytica*** **+** ***E. dispar***		***E. histolytica*** **+** ***E. moshkovskii***	
		**N**	**%**	**n**	**%**	**n**	**%**	**n**	**%**	**n**	**%**
Kampung Gurney	4	4	100	0	0	0	0	0	0	0	0
Kampung Bumbun	12	7	58.3	3	25	0	0	2	16.7	0	0
Kampung Pangsun	10	3	33.3	2	20	0	0	5	50	0	0
Kampung Sungai Minyak	2	2	100	0	0	0	0	0	0	0	0
Kampung Kemensah	2	2	100	0	0	0	0	0	0	0	0
SK Pos Piah	14	11	78.6	0	0	0	0	3	21.4	0	0
Kampung Teras Pos Piah	8	5	62.5	2	25	0	0	1	12.5	0	0
Total	52	34	65.4	7	13.5	0	0	11	21.2	0	0

From the 65 microscope-positive samples, 56 (86.2%) samples were detected by real-time PCR. Of these 56 samples, *E. histolytica* and *E. dispar* mixed infection appeared to be the most predominant (22/56; 39.3%), followed by *E. histolytica* (21/56; 37.5%), *E. dispar* (11/56; 19.6%), and *E. moshkovskii* (1/56; 1.8%). Coexistence of *E. histolytica* and *E. moshkovskii* was identified by real-time PCR in one (1.8%) sample (Table 4).

**Table 4:** **Prevalence of *E. histolytica*, *E. dispar*, and *E. moshkovskii *infections based on real-time PCR in microscopically positive faecal samples according to locations**

**Location**	**Real-time PCR positive**	***E. histolytica***		***E. dispar***		***E. moshkovskii***		***E. histolytica*** **+** ***E. dispar***		***E. histolytica*** **+** ***E. moshkovskii***	
		**n**	**%**	**n**	**%**	**n**	**%**	**n**	**%**	**n**	**%**
Kampung Gurney	5	2	40	0	0	0	0	3	60	0	0
Kampung Bumbun	14	6	42.9	2	14.3	0	0	6	42.9	0	0
Kampung Pangsun	10	2	20	2	20	0	0	5	50	1	10
Kampung Sungai Minyak	2	2	100	0	0	0	0	0	0	0	0
Kampung Kemensah	1	1	100	0	0	0	0	0	0	0	0
SK Pos Piah	16	5	31.3	6	37.5	0	0	5	31.3	0	0
Kampung Teras Pos Piah	8	3	37.5	1	12.5	1	12.5	3	37.5	0	0
Total	56	21	37.5	11	19.6	1	1.8	22	39.3	1	1.8

Kampung Bumbun recorded the highest prevalence of *E. histolytica* (6/56; 10.7%), followed by SK Pos Piah (5/56; 8.9%), Kampung Teras Posh Piah (3/56; 5.4%), Kampung Gurney, Kampung Pangsun, and Kampung Sungai Minyak (2/56; 3.6%) and Kampung Kemensah which recorded only one case of (1/56: 1.8%) *E. histolytica* infection. The highest prevalence of *E. dispar* was found in SK Pos Piah (6/56: 10.7%). As for *E. moshkovskii*, one (1/56: 1.8%) infection was recorded in Kampung Teras Pos Piah. All villages except Kampung Sungai Minyak and Kampung Kemensah recorded a mixed infection of *E. histolytica* and *E. dispar* while a mixed infection of *E. histolytica* and *E. moshkovskii* was only found in Kampung Pangsun.

The real-time PCR and nested PCR are 100% specific as no amplification of other genomic DNA (i.e. in faecal samples of other protozoans and samples negative for parasitic infections) was observed.

There were 9 microscope-positive samples which were negative when tested by real-time PCR as opposed to 13 samples which were negative when tested by nested PCR. PCR tests were repeated for these negative samples but results remained unchanged. Real-time PCR had an overall sensitivity of 86.2% whereas nested PCR reported an overall sensitivity of 80%. Results from the real-time PCR and nested PCR are presented in a 2 × 2 table (Table 1). A Cohen’s kappa value of 0.88 indicated good agreement between the two methods [24]. The McNemar test value showed no significant difference between the two methods (p = 0.221).

### Detection limits

**Figure 1:**
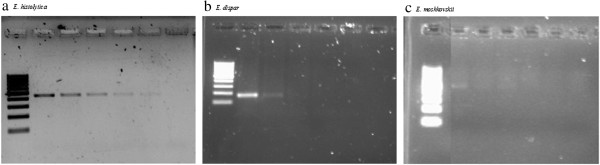
**Results of the polymerase chain reaction (PCR) amplification of a) *E. histolytica*, b) *E. dispar *and c) *E. moshkovskii *with different volume of cysts.** Lane 1, molecular weight marker; Lanes 2–6, amplification with 10, 5, 2.5, 1.25 and 0.625 cysts respectively (2 fold serial dilutions were performed); Lane 7, negative control.

**Figure 2:**
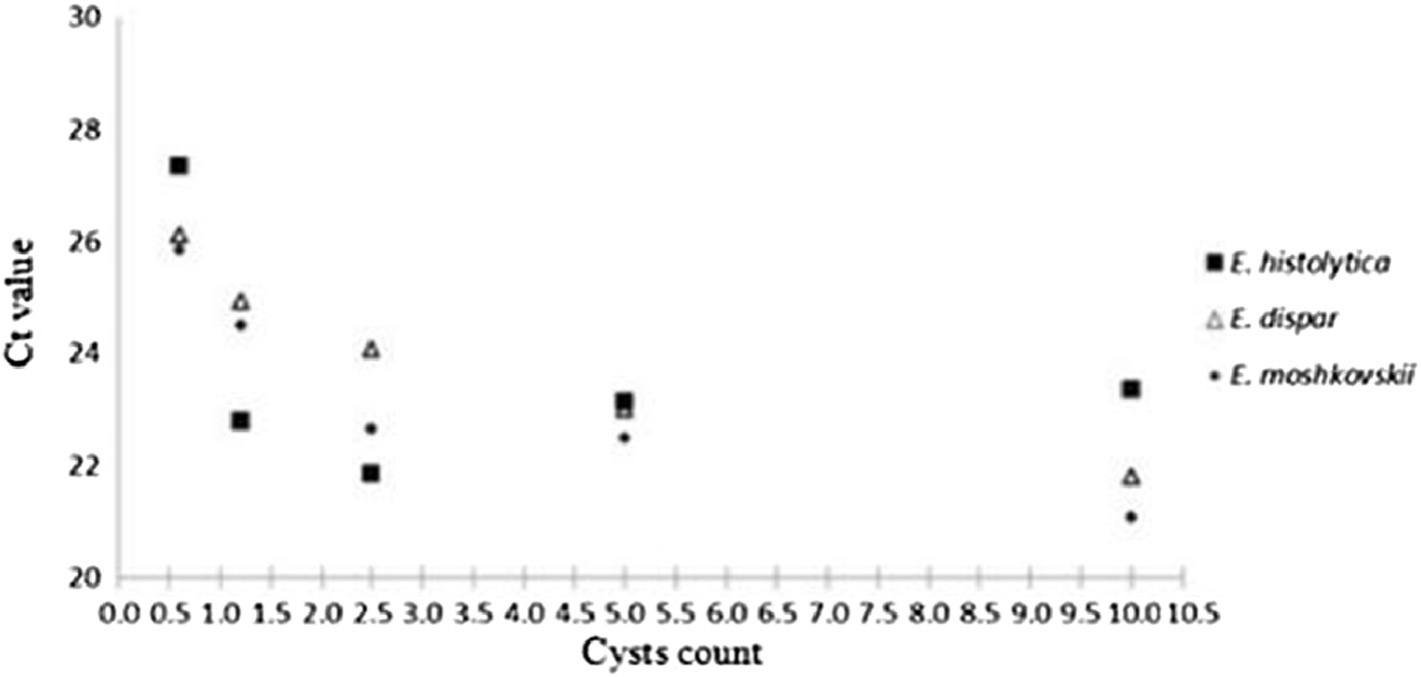
**Detection limits of *Entamoeba *sp. using Real-time PCR.** Real-time PCR showed the detection limits of each *Entamoeba* sp. to be as low as 0.625 cysts.

Detection limits of nested PCR are indicated in Figures 1 and 2. Figure 1 highlights the limitations of nested PCR in identifying *E. dispar* and *E. moshkovskii* infections whereby a minimum of 5 cysts and 10 cysts was required to pick up the respective infections. Although a very faint band was observed, nested PCR was able to identify *E. histolytica* with a cyst count as low as 0.625. The detection of all three *Entamoeba* species by real-time PCR was found to be as low as 0.625 cysts (Figure 2).

## Discussion

The present study reported an overall prevalence of *Entamoeba* species as determined by microscopy to be 19.5% (65/334). This clearly indicates that *Entamoeba* spp. are still very much present in the Orang Asli communities in Malaysia. This reported prevalence is in agreement with previous studies carried out among Orang Asli communities in Malaysia which reported a prevalence rate between 18.5% and 22.5% [25,26].

SK Pos Piah recorded the highest *Entamoeba* prevalence (19/30; 63.3%) in this study. It was found that this aborigine settlement was the farthest away from urban civilization compared to the other settlements which were studied. Poverty, poor socioeconomic conditions, impoverished sanitation and hygiene conditions as well as a lack of education are known factors which contribute to high prevalence of *Entamoeba* infection [27]. Given the location of SK Pos Piah, these contributing factors bring about greater repercussions.

It was noted that most of the settlements in this study did not have toilet facilities, which then led the villagers to defecate near the rivers. Water sources may be contaminated with cysts of parasites from human faeces which are responsible for transmission [28].

Real-time PCR, compared to conventional PCR has several advantages: eliminating the need of post-amplification analysis, which reduces risks of contamination; the ability to better differentiate *E. histolytica*, *E. dispar*, and *E. moshkovskii* infections; and numerical results which are much easier to interpret than the visualization of a stained gel from conventional PCR [3,10]. A noteworthy aspect of real-time PCR is its heightened sensitivity as opposed to conventional PCR. As expected, the real-time PCR assay in this study was more sensitive than the conventional PCR, a finding that is in accordance with a study, which compared real-time PCR assay with conventional PCR for amebiasis [29]. In this study, both probe and primer were manually designed for the identification of *E. moshkovskii*. The present study showed that real-time PCR was able to identify and characterize 56 samples as opposed to 52 samples by nested PCR. This can be explained by the greater detection limit of real-time PCR compared to that of nested PCR. The detection limit of real-time PCR in this study was as low as 0.625 cysts for all three *Entamoeba* species. For nested PCR, a minimum of 5 and 10 cysts were required for the detection of *E. dispar* and *E. moshkovskii* respectively. Only *E. histolytica* could be detected by nested PCR with a cyst count as low as 0.625. The greater sensitivity of real-time PCR (86.2%) over nested PCR (80%) in this study is in agreement with previous studies conducted using real-time PCR [12,26]. In this study, the real-time PCR probe for *E. moshkovskii* was designed manually as aforementioned. A previous study has shown the development of a real-time PCR assay for the detection of *E. moshkovskii*[12]. However, in that study, there was a need to perform the melting curve analysis to differentiate *E. histolytica* and *E. dispar* infections as both infections were detected by the Ehd-640 labeled probe. The advantage of our study is that we were able forego the melting curve analysis since individual probes were used for detection of each of the three *Entamoeba* infections. This significantly reduces the time required in analyzing the real-time PCR results.

The conventional nested PCR assay detected the highest number of *E. histolytica* infection (65.4%), followed by *E. histolytica* and *E. dispar* mixed infection (21.2%), and *E. dispar* (13.5%). In this study, *E*. *moshkovskii* was not detected by nested PCR. In a Malaysian study conducted in 2006, the authors found the prevalence of *E. histolytica* to be greater than that of *E. dispar*, accounting for 13.2% and 5.6% of the *Entamoeba* infection respectively [30]. The higher prevalence of *E. histolytica* is of interest because *E. dispar* is likely to be 10 times more common with reference to the worldwide distribution of *Entamoeba* species [31]. Real-time PCR assay however, found *E. histolytica* and *E. dispar* mixed infection to be most common in this study (39.3%), followed by *E. histolytica* (37.5%), *E. dispar* (19.6%), and *E. moshkovskii* (1.8%). There was one (1.8%) mixed infection of *E. histolytica* and *E*. *moshkovskii* identified by real-time PCR. Detection of *E. moshkovskii* in Malaysia was previously reported by Ngui and colleagues [20] and Shahrul Anuar and co-workers [32] whereby a prevalence rate of 5.8% and 12.3% were recorded respectively using nested PCR [20,32]. The location of study area as well as different Orang Asli ethnic groups could be contributing factors to the large difference in *E. moshkovskii* prevalence rates between this study and the previous studies. Most *E*. *moshkovskii* cases reported worldwide are commonly reported as a mixed infection [31]. Coexistence of *E. histolytica* and *E*. *moshkovskii* has been previously identified in Australia using nested PCR [33].

The nine samples which were negative for real-time PCR were retested and were still found to be negative. It is a possibility that the samples which were detected positive by microscopy but not PCR may belong to other *Entamoeba* species such as *E. coli*, *E. polecki* and *E. hartmanni*[20,31]. A study done by Petri and co-workers [31] in Bangladesh showed the limitations of microscopy, whereby only 40% of children diagnosed by microscopy were proven to have *E. histolytica* infection when compared to PCR.

One of the limitations of this study is that the prevalence of *Entamoeba* was based on a single faecal sample. Due to the fact that many cysts and ova are excreted irregularly, it is recommended that at least three samples are examined for the presence of parasites [34]. In a study conducted by Cartwright [35], it was found that the positivity rate was 55% for patients who had three faecal samples examined as opposed to 33% and 20% for patient who had two and single faecal specimens examined respectively. Unfortunately, obtaining more than one faecal specimen was not possible in this study due to limited resources and also the cultural belief of the aborigines which is against the giving of their faecal specimens. This was mentioned in a study done by Anuar and colleagues [18].

As far as we know, this is the first study using real-time PCR assay in Malaysia to differentiate between the three *Entamoeba* species. It would be noteworthy to consider real-time PCR as an alternative tool in epidemiological studies and the diagnosis of amebiasis as this method will provide epidemiological data that is more accurate as well as establish a better understanding of *Entamoeba* infections.

## Conclusions

As far as we know, this is the first study using real-time PCR assay in Malaysia to differentiate between the three *Entamoeba* species. It would be noteworthy to consider real-time PCR as an alternative tool in epidemiological studies and the diagnosis of amebiasis as this method will provide epidemiological data that is more accurate as well as establish a better understanding of *Entamoeba* infections.

## Competing interests

The authors declare that they have no competing interests.

## Authors’ contributions

LYL, JI and RM took part in the conception, planned and designed the protocols. SAF performed laboratory work and conducted the field study, including the collection of stool samples and data from the questionnaire interviews. CA performed laboratory work and prepared the manuscript. LYL reviewed the drafts of the manuscript for important intellectual content. All authors have seen and approved the final version of the manuscript.
